# Novel chemical stimulation for geothermal reservoirs by chelating agent driven selective mineral dissolution in fractured rocks

**DOI:** 10.1038/s41598-021-99511-6

**Published:** 2021-10-07

**Authors:** Noriaki Watanabe, Kaori Takahashi, Ryota Takahashi, Kengo Nakamura, Yusuke Kumano, Kohei Akaku, Tetsuya Tamagawa, Takeshi Komai

**Affiliations:** 1 Department of Environmental Studies for Advanced Society, Graduate School of Environmental Studies, Tohoku University, Sendai, 9808579 Japan; 2 Technical Division, Research Center, Japan Petroleum Exploration Co., Ltd. (JAPEX), Chiba, 2610025 Japan

**Keywords:** Geochemistry, Hydrogeology

## Abstract

Improving geothermal systems through hydraulic stimulation to create highly permeable fractured rocks can induce seismicity. Therefore, the technique must be applied at a moderate intensity; this has led to concerns of insufficient permeability enhancement. Adding chemical stimulation can mitigate these issues, but traditional methods using strong mineral acids have challenges in terms of achieving mineral dissolution over long distances and highly variable fluid chemistry. Here, we demonstrate a novel chemical stimulation method for improving the permeability of rock fractures using a chelating agent that substantially enhances the dissolution rate of specific minerals to create voids that are sustained under crustal stress without the challenges associated with the traditional methods. Applying this agent to fractured granite samples under confining stress at 200 °C in conjunction with 20 wt% aqueous solutions of sodium salts of environmentally friendly chelating agents (*N*-(2-hydroxyethyl)ethylenediamine-*N*, *N*′, *N*′-triacetic acid and *N, N*-bis(carboxymethyl)-l-glutamic acid) at pH 4 was assessed. A significant permeability enhancement of up to approximately sixfold was observed within 2 h, primarily due to the formation of voids based on the selective dissolution of biotite. These results demonstrate a new approach for chemical stimulation.

## Introduction

Geothermal energy, a renewable energy source, can be used to generate baseload electricity. However, to date, conventional geothermal power generation facilities have been operated only in regions with high geothermal gradients and reservoir permeability. Enhanced geothermal systems (EGSs) are currently being developed to enable geothermal power generation from reservoirs with sufficiently high temperatures but low initial permeabilities, such as granitic rock at significant depths in non-volcanic areas ^1,2^.

An EGS requires the initial low reservoir permeability to be increased using hydraulic, thermal, and/or chemical stimulation^3–10^. Among these methods, hydraulic stimulation that induces shear dilation of pre-existing fractures and/or creates new fractures via the injection of high-pressure fluids has been frequently used and widely researched. Despite this, the possibility of inducing seismic events by hydraulic stimulation is a concern^11–14^; therefore, this technique should be used carefully in conjunction with injection flow rate and pressure of moderate intensities^15,16^. However, lowering the intensity of the procedure leads to concerns related to insufficient permeability enhancement.

It is known that the induced shear slip and dilation (i.e. shear dilation) of pre-existing fractures by the injection of high-pressure fluids is a part of the primary effects that enhance reservoir permeability during hydraulic stimulation. Consequently, several researchers argue that hydraulic stimulation should be conducted at injection pressures below the minimum principal stress and that self-propped fractures associated with asperities are an effective means of retaining permeability^1,17–20^. Even then, the creation of new fractures could be an integral part of the shear stimulation process because shear slip increases the stress intensity at the tips of pre-existing fractures, potentially leading to fracture propagation to generate fracture networks^21^. However, a significantly enhanced fracture permeability does not necessarily accompany shear slip.

Laboratory experiments involving the injection-induced shear slip of fractures in granite have demonstrated permeability enhancement factors (i.e. ratios of permeability after and before shear slip) ranging from 0 to approximately 4 based on shear slips of less than 1 mm^22^. Moreover, the new fractures created in this process may not be highly permeable because such fractures do not always occur in a stress state that favours shear slip (i.e. shear dilation), although they can still contribute to the formation of a fracture network^21^. If the increased permeability is insufficient after a hydraulic stimulation trial, greater enhancement can be achieved following the deformation of the reservoir as larger shear slips of initially large or coalesced fractures are induced by subsequent longer and/or higher pressure injections^23–27^, but additional injections can increase the risk of inducing seismicity. Balancing the conflicting concerns of insufficient permeability enhancement and induced seismicity from hydraulic stimulation requires additional stimulation methods, such as chemical stimulation.

Chemical stimulation techniques, the cost of which is lower than hydraulic stimulation, consist mainly of injecting acid into the reservoir to remove damaged regions near the wellbore resulting from hydrothermal minerals (e.g., carbonates, feldspars, and micas) deposited in fractures and/or the creation of larger aperture fractures via the dissolution of rock-forming minerals instead of shear dilation^3,8–10^. However, it is well known that with conventional acidizing techniques using hydrochloric and hydrofluoric acids, it is difficult to dissolve over long distances. For instance, numerical simulations of chemical stimulation using hydrochloric acid in the EGS of Soultz-sous-Forêts (Alsace, France) have been studied^10^. Here, the stimulation fluid is injected at a low temperature (65 °C) to the granitic reservoir at 200 °C, and it was demonstrated that the stimulated distance from the injection well is less than 3 m in all tested combinations of the injection rate, injection duration, and acid concentration. It is also difficult to predict all the dissolution and precipitation reactions that can occur and the resulting permeability changes because of the exceptionally high reactivity of minerals and the highly variable fluid chemistry.

Because of such difficulties related to the use of conventional acids, chelating agents have been used in the stimulation of carbonate and sandstone hydrocarbon reservoirs^28,29^, even though chelating agents are more expensive than conventional acids. The dissolution rate of minerals in rocks can be sufficiently high even when using weakly acidic aqueous solutions if chelating agents are used in addition to hydrogen ions to attack the rock^30^. Increased dissolution rates and metal ion stabilisation by chelation (i.e. formation of stable water-soluble metal-chelate complex) over longer distances are the main reasons for using chelating agents instead of conventional acids in the stimulation of hydrocarbon reservoirs.

Among the various kinds of chelating agents, environmentally friendly compounds such as *N*-(2-hydroxyethyl)ethylenediamine-*N*, *N′*, *N′*-triacetic acid (HEDTA) and *N, N*-bis(carboxymethyl)-l-glutamic acid (GLDA) are well suited to chemical stimulation^31,32^. A previous study examined the use of HEDTA solution of pH 2.5, 4, and 9 to stimulate carbonates at approximately 150 °C, and reported that the use of HEDTA at pH 4 was effective^33^. Another study examined GLDA, which is readily biodegradable, and compared it with other chelating agents, including HEDTA. This previous study reported that GLDA is as effective as other chelating agents and has the same degree of thermal stability as HEDTA^34^. A recent study regarding the thermal stability of major chelating agents, including GLDA and HEDTA, suggested that these agents can be used at temperatures of up to approximately 200 °C^35^. These previous studies have established the potential applications of environmentally friendly chelating agents to the chemical stimulation of geothermal reservoirs, especially for reservoirs with temperatures of up to 200 °C.

Herein, we propose a novel chemical stimulation technique using chelating agents to increase the permeability of rock fractures through the selective dissolution of rock-forming minerals in geothermal environments. This process has been primarily designed for use at reservoir temperatures of up to 200 °C. However, it may be used at higher reservoir temperatures by employing a cold fluid injection to satisfy an operable temperature limit while expecting that various thermal stimulation effects will open small aperture fractures and/or create thermal fractures to make the chemical stimulation easier and more effective^4^. Under any stimulation scenario, the temperature of the stimulation fluid and therefore the reaction rate may increase with increasing distance from the injection well because the temperature of the injection well will be lower than the reservoir temperature. This may reduce spatially uneven stimulations because the reaction rate is lower near the injection well in which the rocks are exposed to the stimulation fluid for a longer time. Selective mineral dissolution is desirable because it simplifies the permeability evolution mechanism and creates voids within a framework of less soluble minerals that remain open under confining stress at depth.

To the best of our knowledge, the present study is the first to examine the fundamental characteristics of a novel chemical stimulation using weakly acidic (pH 4) solutions of the environmentally friendly chelating agents HEDTA and GLDA. Although GLDA may be better suited to this application because of its ready biodegradability, HEDTA was also assessed not to constrain this new method based on the availability of a specific chelating agent. Initial tests were performed to examine the flooding of fractured granite under confining stress at 200 °C. For granite consisting primarily of quartz (ideal chemical composition: SiO_2_), K-feldspar (ideal chemical composition: KAlSi_3_O_8_), plagioclase (ideal chemical compositions of the solid-solution end-members: NaAlSi_3_O_8_ and CaAl_2_Si_2_O_8_), and biotite (ideal generalized chemical composition: K(Mg,Fe)_3_AlSi_3_O_10_(OH,F)_2_), the dissolution rates of minerals containing Al, Ca, Fe, or Mg (i.e. minerals other than quartz) were expected to be enhanced by the addition of chelating agents. This enhancement was anticipated to be different for different types of minerals. The results of these tests are presented herein and the mechanism and viability of selective mineral dissolution by chelating agents are discussed.

## Chelating agent flooding experiments

### Fractured granite samples and chelating agents

**Figure 1:**
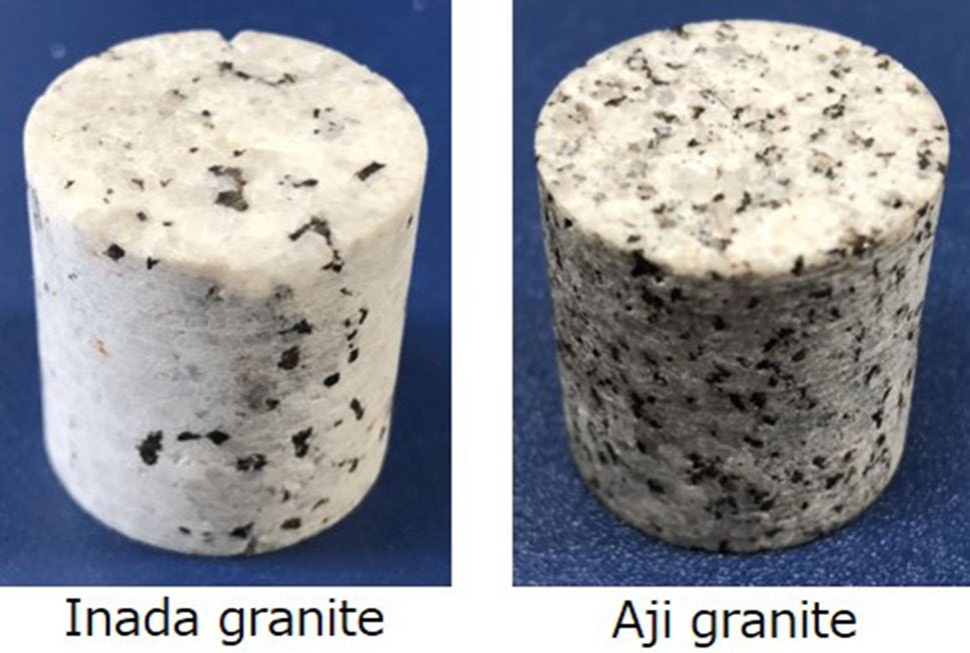
Two types of granite used in chelating agent flooding experiments.

Four cylindrical samples (diameter: 25 mm, length: 25 mm) were prepared using two types of granite, containing either a single fracture induced by the Brazilian test or multiple fractures induced by heating in an electric furnace at 570 °C and atmospheric pressure for 2 h. Coarser-grained Inada granite and finer-grained Aji granite (Fig. 1), from Ibaraki and Kagawa prefectures, Japan, respectively, were used to prepare two samples containing multiple fractures (Inada granite), and the other two samples containing a single fracture (Inada granite and Aji granite, respectively). Inada granite and Aji granite mainly consist of quartz, K-feldspar, plagioclase, and biotite with small quantities of hornblende (ideal generalized chemical composition: Ca_2_(Mg,Fe)_4_Al(AlSi_7_O_22_)(OH)_2_)^36–39^. Table 1 explains the large difference in the average grain size and similarity in the mineral composition between the two types of granite^37,39^. X-Ray computed tomography (CT) images were acquired from all samples before and after chelating agent flooding experiments at a tube voltage of 120 kV, tube current of 150 μA, and voxel size of 20 μm × 20 μm × 20 μm. Molcer Plus 3D image visualisation and processing software (White Rabbit Corp.)^40^ was used to visualise the distributions of significantly large pores and fracture apertures (i.e. voids) with sizes similar to or larger than the voxel size.

**Table 1:** Average grain size and mineral composition of the two types of granite.

Type of granite	Average grain size (mm)	Mineral content (vol%)				
		Quartz	K-feldspar	Plagioclase	Biotite	Others
Inada	2.8	33.7	32.1	30.3	3.8	0.1
Aji	0.5	29.4	26.4	37.6	6.5	0.1

Weakly acidic aqueous solutions containing either GLDA or HEDTA were prepared from 40.0 wt%–44.0 wt% aqueous solutions of GLDA-Na_4_ (C_9_H_9_NNa_4_O_8_) or HEDTA-Na_3_ dihydrate (C_10_H_15_N_2_Na_3_O_7_·2H_2_O) powder (> 98.0% purity), purchased from Tokyo Chemical Industry Co., Ltd. Here, the price per unit weight of HEDTA-Na_3_ dihydrate is estimated to be approximately one-fourth that of GLDA-Na_4_. These solutions contained either GLDA-Na_4_ or HEDTA-Na_3_ at approximately 20 wt% and were adjusted to a pH of 4 with nitric acid, which mimics a commercially used GLDA-based stimulation fluid for hydrocarbon reservoirs (Dissolvine^®^ StimWell provided by Nouryon N.V.). It should be noted that the acid for pH adjustment is not limited to nitric acid. In the present study, nitric acid, which does not corrode stainless steel, was employed in preparation for an unexpected accident originating from corrosion of stainless-steel parts used in our experimental system, even though we expected such an accident would not occur under such weakly acidic conditions.

### Experimental system, procedures, and conditions

**Figure 2:**
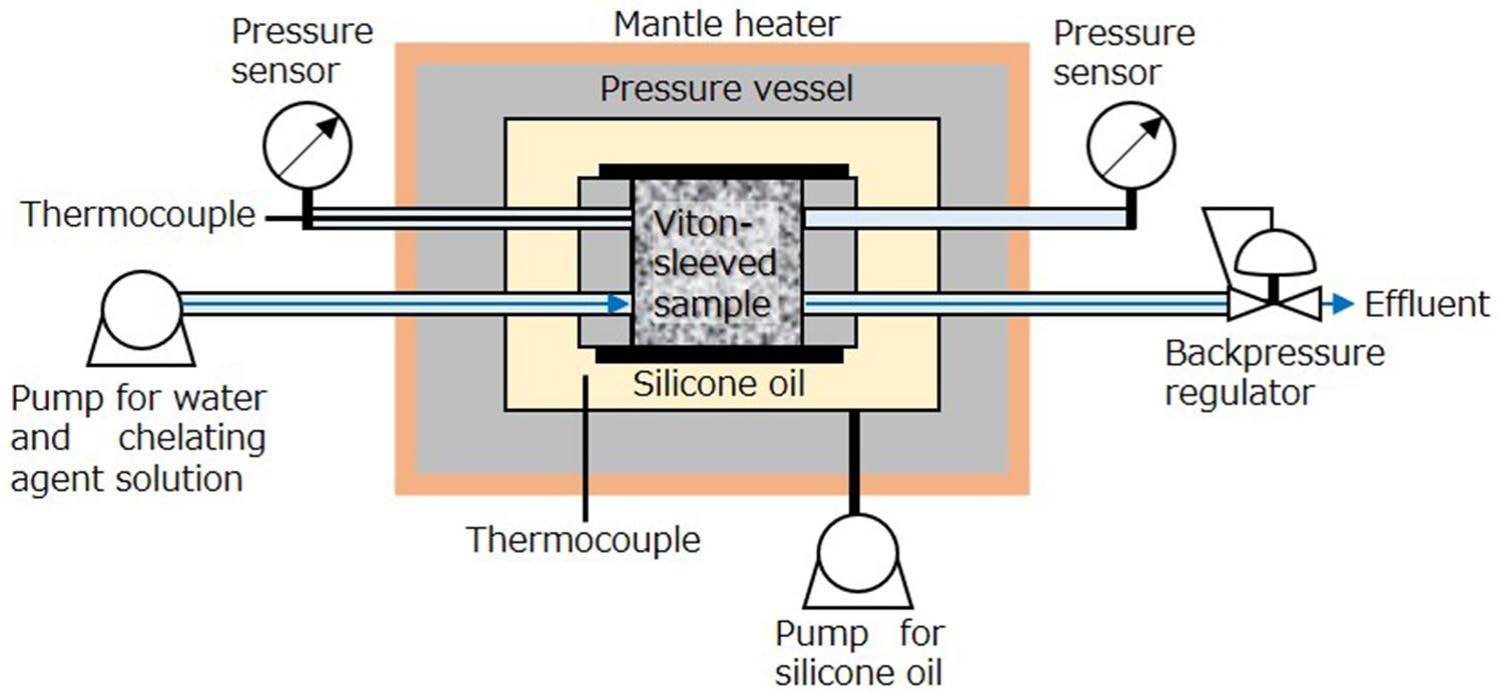
Experimental system for chelating agent flooding experiments.

The chelating agent flooding experiments were conducted using the experimental system displayed in Fig. 2. In each trial, a Viton-sleeved sample, with end plugs attached at both inlet and outlet faces, was placed horizontally in a pressure vessel maintained at 200 °C using a mantle heater. The sample was subjected to confining stress of 15 MPa, representing confining stress in geothermal environments, by pumping silicone oil into the vessel at a constant pressure. The initial permeability of the sample was determined based on the Darcy’s law by first injecting pure water into the sample at a constant flow rate of either 0.25 or 1.00 mL min^−1^, depending on sample permeability, using a pump. The permeability (*k*) can be determined based on the Darcy’s law:

1 $$ k = \frac{Q\mu L}{{\pi r^{2} \Delta P}}, $$

where *Q* is the flow rate, *μ* is the viscosity of the injected fluid, Δ*P* is the differential pressure for the fluid between the inlet and outlet faces of the sample, and *r* and *L* are the radius and length of the sample, respectively. In this process, the water flowed out of the sample through a backpressure regulator adjusted to 5 MPa. After this initial permeability assessment, the aqueous chelating agent solution was injected into the sample at the same flow rate for 2 h. Effluent samples were collected at constant intervals of either 10 min (at 1.00 mL min^−1^) or 20 min (at 0.25 mL min^−1^) throughout each experiment. These aliquots were analysed to determine the concentrations of Al, Ca, Fe, K, Mg, and Si using inductively coupled plasma optical emission spectrometry (ICP-OES).

Four experiments were conducted during this first-ever evaluation of chelating agent flooding through fractured granite under geothermal conditions. The combinations of sample type, chelating agent, and injection rate used in each trial are summarised in Table 2. The first and second experiments (Runs 1 and 2) were conducted using the coarser-grained Inada granite with multiple fractures, employing HEDTA and GLDA, respectively, to confirm the increased permeability of the fractured granite via the expected selective mineral dissolution induced by the chelating agents. Samples with multiple fractures were examined initially because it was expected that it would be easier to identify the differences in X-ray CT images taken before and after the experiment. This was because of the larger number of contact locations between the chelating agent solution and minerals in the samples. The third experiment (Run 3) was conducted using the coarser-grained Inada granite with a single fracture and GLDA as the chelating agent. The purpose was to confirm that the permeability enhancement was caused by selective mineral dissolution solely in fractures rather than in both fractures and bulk matrix. The fourth experiment (Run 4) was conducted with finer-grained Aji granite with a single fracture, and GLDA was used to examine the effect of grain size on permeability enhancement, although it may not have been a complete examination because it was not possible to use two types of granite with the same mineral content but different grain sizes (Table 1).

**Table 2:** Granite samples, chelating agents, and injection rates in each experiment.

Experiment	Granite sample	Chelating agent	Injection rate (mL min^−1^)
Run 1	Coarser-grained Inada granite with multiple fractures	HEDTA	1.00
Run 2		GLDA	
Run 3	Coarser-grained Inada granite with a single fracture	GLDA	0.25
Run 4	Finer-grained Aji granite with a single fracture	GLDA	0.25

## Results and discussion

### Viability of new chemical stimulation process

**Figure 3:**
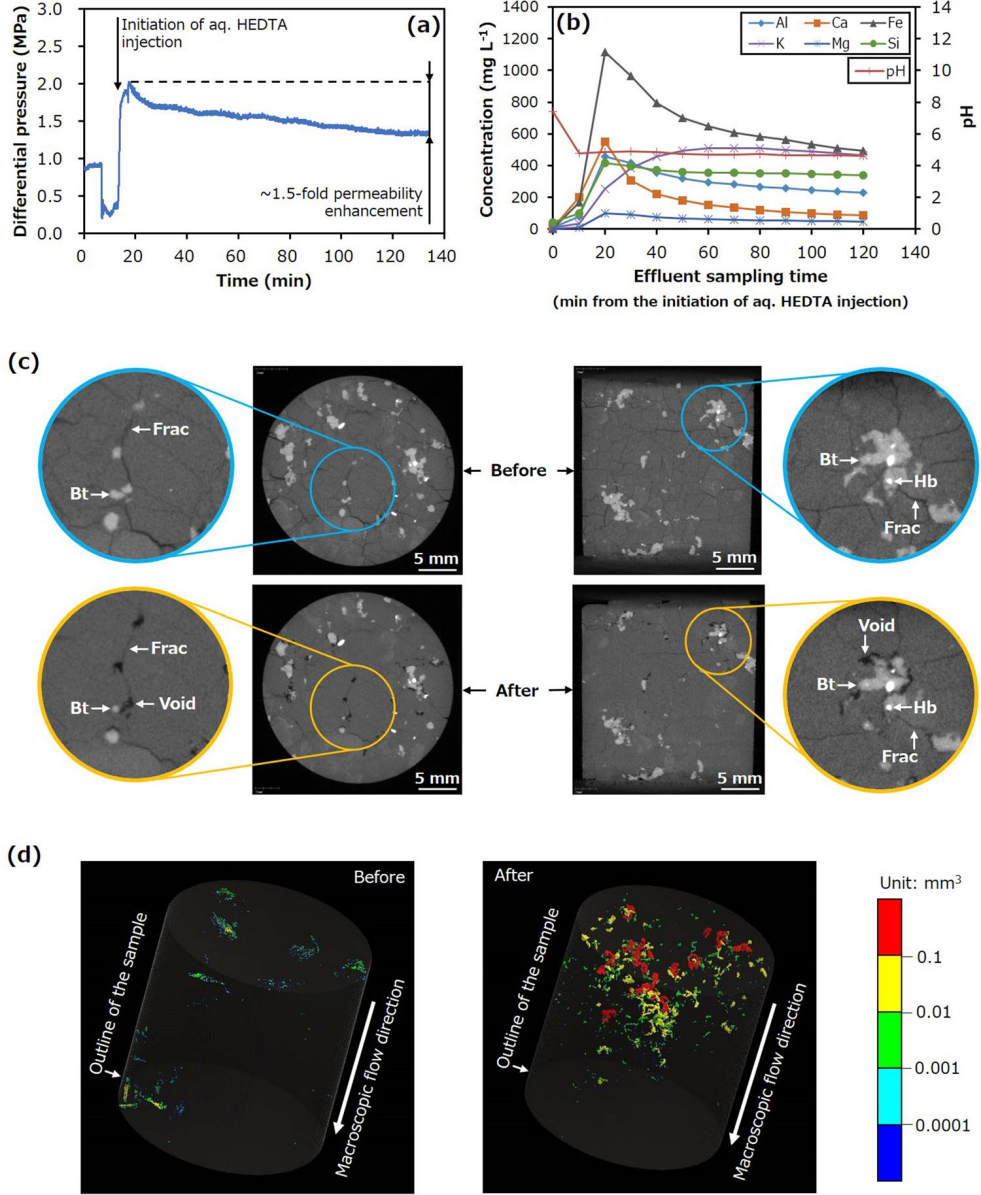
Hydraulic and chemical behaviours, and X-ray CT images for Run 1. (**a**) Differential pressure between the inlet and outlet as a function of time and (**b**) concentrations of various elements in the effluent and effluent pH as functions of time during Run 1. X-ray CT images displaying (**c**) the sample before and after the trial and (**d**) distributions of significantly large pores and fracture apertures before and after the trial (*Bt* Biotite, *Hb* Hornblende, *Frac* Fracture).

The possibility of increasing the permeability of the fractured granite by chelating-agent-driven selective mineral dissolution was confirmed by the first experiment (Run 1). Figure 3 is a plot of the differential pressure between the inlet and outlet (proxy of hydraulic resistance based on the Darcy’s law represented by Eq. (1)) and the elemental concentrations and pH of the effluent as a function of time, where the concentration and pH at 0 min were obtained for the effluent before injecting the chelating agent solution. This figure also provides X-ray CT images displaying the sample and the distribution of voids in the sample before and after the trial. It should be noted that these images were selected to compare very similar slices, although they may not precisely present the same slices. Here, hornblende and biotite (the minerals with the lowest and second-lowest contents, respectively) appear as white and light grey regions, whereas the other minerals (quartz, K-feldspar, and plagioclase) appear as similar dark grey zones. Additionally, fractures and pores appear as darker grey and black areas, respectively. It should be noted that the induced fractures that appeared after the experiments tended to have smaller apertures even if no chemical reaction occurred because they had been subjected to confining stress. For the voids in these images, warmer colours correspond to larger sizes.

The differential pressure was almost constant during the water injection process and indicated an initial sample permeability of approximately 1.0 × 10^–16^ m^2^ (Fig. 3a). In contrast, after initiating the injection of the HEDTA solution, the differential pressure first increased and then continually decreased. It should be noted that the temporal decrease in differential pressure before injecting the chelating agent solution was caused by stopping the pump to switch the fluid from water to chelating agent solution because the same pump was used for both fluids. The initial increase in the differential pressure can be attributed to the higher viscosity of the chelating agent solution, whereas the continuous decrease can be ascribed to the continuous enhancement of sample permeability. The difference between the peak and final values during the HEDTA solution injection demonstrates that the permeability was increased by a factor of approximately 1.5, within 2 h. This result is significant because the permeability enhancement factors obtained from the injection-induced shear slip of fractures in granite in previous laboratory experiments ranged from 0 to approximately 4^22^.

The concentrations of all elements in the effluent increased significantly after HEDTA solution injection, demonstrating the expected enhanced dissolution of various minerals by the weakly acidic chelating agent solution. It should be noted that this increased dissolution was not observed in a preliminary experiment using a buffered solution (pH 4.0) without a chelating agent. Prior work has demonstrated that enhanced dissolution of minerals other than quartz may occur at much lower pH values^41–45^ but is unlikely at the pH of 4 used in the present study.

During injection of the chelating agent solution, the concentrations of Al, Ca, Fe, and Mg initially increased, and then gradually decreased, whereas the concentrations of Si and K initially increased and then became approximately constant (Fig. 3b). These trends may reflect the enhanced dissolution of several minerals in the sample, including K-feldspar, plagioclase, biotite, and hornblende. However, the changes in the Fe and Mg concentrations are likely related mainly to the dissolution of biotite because hornblende was present at a considerably lower content. The occurrence of peak Fe and Mg concentrations can be attributed to the high spatial heterogeneity of the biotite dissolution process, resulting from much higher reactivity of the edges than that of the basal planes in this material^42^.

The X-ray CT images established the formation of voids due to the selective dissolution of biotite (Fig. 3c), and the distribution of these voids were formed at various specific locations (Fig. 3d). It should be noted that several of the biotite grains dissolved completely, as shown in the left images in Fig. 3c, suggesting that the formation of a leached layer or secondary mineral (i.e. formation of residue) during biotite dissolution may have been insignificant if present. Consequently, permeability enhancement was primarily caused by the selective dissolution of biotite to produce these voids. Although the enhanced dissolution of various minerals occurred during these trials, biotite dissolved to a considerably greater extent than the other minerals, resulting in selective mineral dissolution to form voids sustained under confining stress. The formation of voids via the selective dissolution of biotite in this manner was unexpected because this phenomenon has not been reported previously.

**Figure 4:**
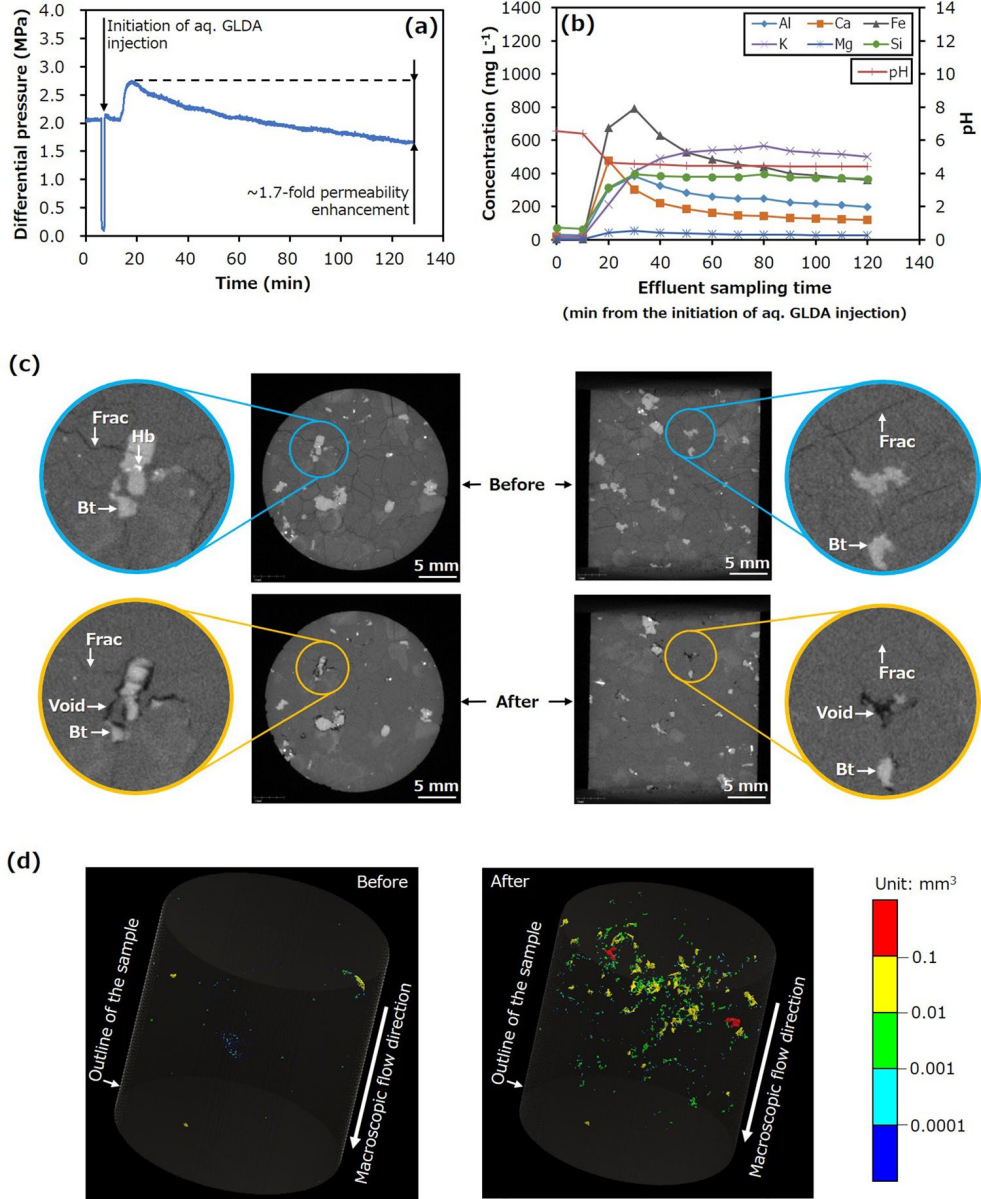
Hydraulic and chemical behaviours, and X-ray CT images for Run 2. (**a**) Differential pressure between the inlet and outlet as a function of time and (**b**) concentrations of various elements in the effluent and effluent pH as functions of time during Run 2. X-ray CT images displaying, (**c**) the sample before and after the trial and (**d**) distributions of significantly large pores and fracture apertures before and after the trial (*Bt* Biotite, *Hb* Hornblende, *Frac* Fracture).

Similar results were also observed in the second experiment (Run 2), during which the only difference in the experimental conditions from those in Run 1 was a change in the chelating agent to GLDA. Figure 4 plots the associated changes in the differential pressure, elemental concentrations, and pH alongside the X-ray CT images. The initial permeability of approximately 7.0 × 10^–17^ m^2^ was observed to increase by a factor of approximately 1.7 (Fig. 4a), mainly due to the formation of voids as a result of biotite dissolution (Fig. 4b–d). Consequently, the results from Runs 1 and 2 demonstrate that the permeability of fractured granite can be enhanced significantly and rapidly under geothermal conditions, primarily by the selective dissolution of biotite in response to the application of a chelating agent, including the readily biodegradable compound GLDA.

### Characteristics of chemical stimulation process

**Figure 5:**
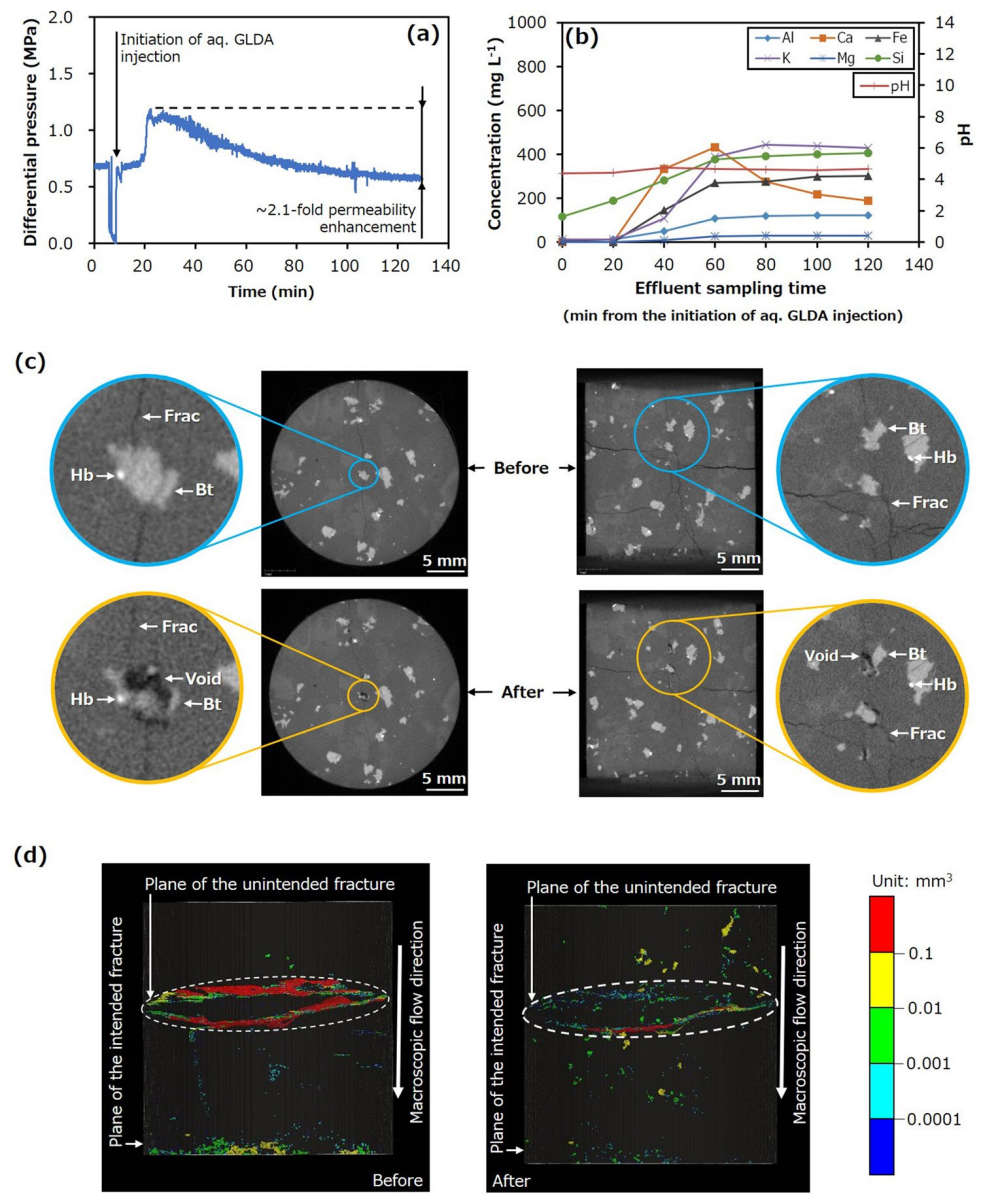
Hydraulic and chemical behaviours, and X-ray CT images for Run 3. (**a**) Differential pressure between the inlet and outlet as a function of time and (**b**) concentrations of various elements in the effluent and effluent pH as functions of time during Run 3. X-Ray CT images displaying, (**c**) the sample before and after the trial and (**d**) distributions of significantly large pores and fracture apertures before and after the trial (*Bt* Biotite, *Hb* Hornblende, *Frac* Fracture).

It was unclear from the first two trials if biotite dissolution occurred solely in fractures or in both fractures and bulk matrix because of diffusion of the chelating agent (meaning a loss of the chelating agent intended to stimulate the fracture). To clarify this point, the third experiment (Run 3) was conducted under the same conditions as in Run 2, but with the sample having a single fracture. The changes in the differential pressure, elemental concentrations, and pH over time, along with the X-ray CT images, are presented in Fig. 5. It should be noted again that the induced fractures that appeared after the experiments tended to have smaller apertures even if no chemical reaction occurred because they had been subjected to confining stress.

Interestingly, the pH of the effluent, as shown in Fig. 5b, was relatively low (approximately 4) even before injecting the weakly acidic chelating agent solution. However, this relatively small pH change is considered to have had no significant effect on the present experiment, as demonstrated in the figure. The X-ray CT images acquired before the trial also displayed several unexpected fractures in the sample approximately perpendicular to the macroscopic flow direction during the experiment (Fig. 5c). This sample was used in the trial despite the presence of these fractures because their presence did not affect the processes that were being assessed in the study.

During this trial, a similar change in differential pressure was observed, as in the other trials (Fig. 5a), such that the initial permeability of approximately 4.6 × 10^–17^ m^2^ was increased by a factor of approximately 2.1. However, the changes in the Al, Fe, and Mg concentrations did not exhibit peaks, although the levels of all elements increased after initiating GLDA solution injection (Fig. 5b). This difference may have been caused by the smaller number of fractures in the coarser-grained granite, which reduced the contact between the chelating agent and the highly reactive biotite edges responsible for the release of Fe and Mg (and possibly Al).

The X-ray CT images confirmed biotite dissolution and the appearance of voids only along the fracture and parallel to the macroscopic flow direction (Fig. 5c,d). Thus, it is apparent that advective transport of the chelating agent will proceed in fractured rocks when injecting the chelating agent solution into the reservoirs. This form of transport is desirable because it reduces the loss of chelating agents due to diffusion into hydraulically ineffective pores/fractures, such that hydraulically effective pores/fractures may be stimulated to efficiently form permeable fracture networks.

The increase in permeability apparent from Runs 1 through 3 can be attributed primarily to the formation of voids due to the dissolution of biotite only along existing fractures. Therefore, it is likely that the degree of permeability enhancement is highly dependent on the distribution of biotite within the granite. In this case, the extent of increased permeability in finer-grained granite is likely to be larger than that observed for coarser-grained granite. This would occur because there would be many contacts between the chelating agent and the biotite grains in the finer-grained granite. To examine this hypothesis, the final experiment (Run 4) was conducted under similar conditions to those in Run 3, except that the rock sample was finer-grained granite.

**Figure 6:**
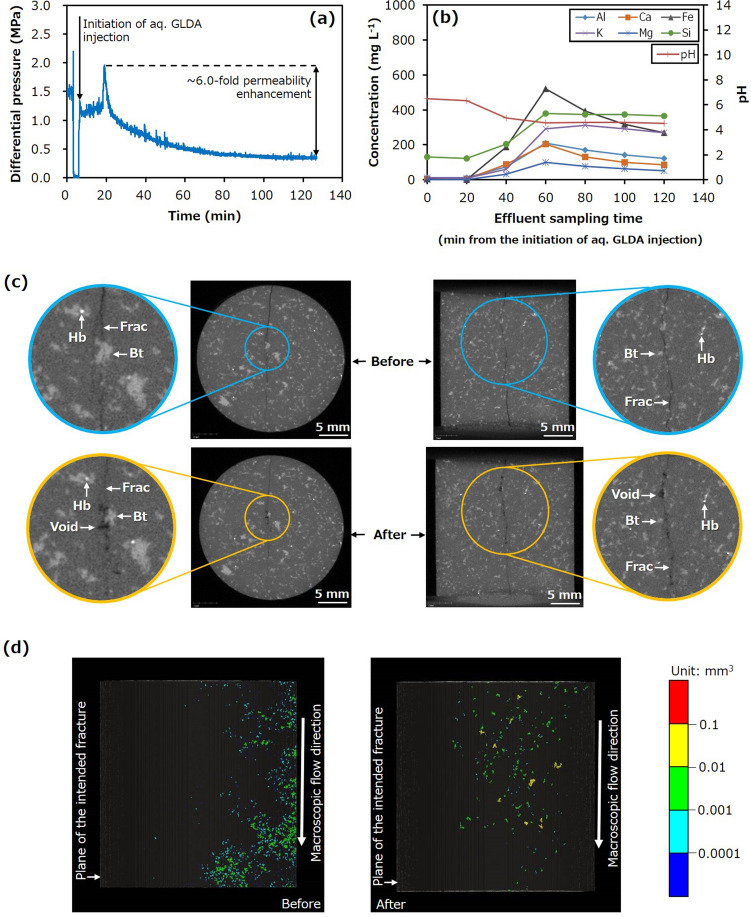
Hydraulic and chemical behaviours, and X-ray CT images for Run 4. (**a**) Differential pressure between the inlet and outlet as a function of time and (**b**) concentrations of various elements in the effluent and effluent pH as functions of time during Run 4. X-ray CT images displaying (**c**) the sample before and after the trial and (**d**) distributions of significantly large pores and fracture apertures before and after the trial (*Bt* Biotite, *Hb* Hornblende, *Frac* Fracture).

Plots of the differential pressure, elemental concentrations, and pH, together with the X-ray CT images, are presented in Fig. 6. The differential pressure decreased much more rapidly and significantly during GLDA solution injection than in the other trials, with an initial permeability of approximately 2.0 × 10^–17^ m^2^ that was increased by a factor of approximately 6.0 (Fig. 6a). Additionally, the formation of voids via the selective dissolution of biotite occurred at a greater number of locations in the fracture plane (Fig. 6b–d), which may have caused the more rapid and more pronounced permeability enhancement. The permeability enhancement in this trial was close to three times that achieved when using the coarser-grained fractured granite in Run 3. This increase in permeability was also superior to that produced by the injection-induced shear slip of fractures in granite in previous laboratory experiments^22^.

These data establish that the proposed chemical stimulation process can efficiently enhance the permeability of fracture networks in granitic geothermal reservoirs. The degree of permeability enhancement can be similar to or larger than that achieved from the injection-induced shear slip of fractures, depending on the distribution of biotite within the reservoir rocks. One concern related to this new chemical stimulation process is the thermal stability of the chelating agents^35^ when a chelating agent is expected to be subjected to temperatures exceeding 200 °C during the stimulation of a reservoir of > 200 °C; however, it is not the primary target. Although we did not observe any adverse effects related to thermal stability in the present experiments at 200 °C, as expected from the reported thermal stability described in the introduction section, there is a concern that the thermal decomposition of the chelating agents and/or metal ions released due to the decomposition may generate deposits in such challenging applications. Therefore, it could be preferable for such challenging applications to employ a flow-back system for the injected chelating agent solution before adverse effects occur but after achieving sufficient stimulation. However, it is unclear whether complete flow-back is feasible in the present stage, because we cannot simulate the new chemical stimulation process in an actual geothermal reservoir. Another concern is the effectiveness of the new chemical stimulation for reservoirs that initially contain acidic brines and therefore altered granitic rocks. Although no adverse influence on permeability is expected to the best of our knowledge, the degree and/or behaviour of permeability enhancement may be different from those observed in the present experiments on unaltered granite. In the future, it will be necessary to assess whether this chemical stimulation should be used in addition to hydraulic stimulation or instead of multiple hydraulic stimulations to meet the dual requirements of sufficiently high reservoir permeability and low risk of induced seismicity. Future investigations employing observations of a higher spatial resolution for fractures and pores in rocks should examine the effects of variables such as rock type, chelating agent, temperature, pH, and stress state, in more detail, to finally allow numerical simulations of the new chemical stimulation in actual geothermal reservoirs.

## Conclusions

The use of hydraulic stimulation to improve geothermal systems has concerns related to induced seismicity; therefore, the technique should be used with utmost care at a moderate intensity, leading to the additional challenge of insufficient permeability enhancement. To address these conflicting concerns, we proposed a novel chemical stimulation method using chelating agents to induce selective mineral dissolution in rock fractures. This process is an alternative to the conventional acidising technique using mineral acids, which can lead to difficulties associated with the exceptionally high reactivity of many minerals and extremely variable fluid chemistry.

The chelating agent flooding experiments performed on fractured granite under confining stress at 200 °C using 20 wt% aqueous solutions of sodium salts of environmentally friendly chelating agents (HEDTA and GLDA) at pH 4 in the present study demonstrate that the permeability of fractured granite can be rapidly and significantly improved under geothermal conditions (e.g., up to approximately six-fold improvement within 2 h under the present experimental conditions). This is primarily the result of the formation of voids due to the selective dissolution of biotite based on the application of environmentally friendly chelating agents, including the readily biodegradable compound GLDA. The results of the present study suggest that the increased permeability of granite reservoirs occurs via selective dissolution mainly in hydraulically effective fractures due to the advective transport characteristics of chelating agents. Additionally, it is also suggested that permeability enhancement is more significant in finer-grained granite because of the selective dissolution of biotite, which has a denser spatial distribution in such rocks.

The present work demonstrates the possibility and effectiveness of the novel chemical stimulation to improve geothermal reservoirs. The use of this novel process in addition to hydraulic stimulation instead of multiple hydraulic stimulations may provide sufficiently high reservoir permeability with a decreased risk of induced seismicity. Future investigations of this new chemical stimulation technique for different conditions are expected to contribute to the extensive use of EGSs worldwide in the future.

## Data Availability

The data that support the findings of this study are available from the corresponding author on reasonable request.
